# Integrative network analysis reveals active microRNAs and their functions in gastric cancer

**DOI:** 10.1186/1752-0509-5-99

**Published:** 2011-06-26

**Authors:** Chien-Wei Tseng, Chen-Ching Lin, Chiung-Nien Chen, Hsuan-Cheng Huang, Hsueh-Fen Juan

**Affiliations:** 1 Institute of Molecular and Cellular Biology and Department of Life Science, National Taiwan University, Taipei 106, Taiwan; 2 Institute of Biomedical Electronics and Bioinformatics, National Taiwan University, Taipei 106, Taiwan; 3 Institute of Biomedical Informatics and Center for Systems and Synthetic Biology, National Yang-Ming University, Taipei 112, Taiwan; 4 Angiogenesis Research Center, National Taiwan University, Taipei 106, Taiwan; 5 Department of Surgery, National Taiwan University Hospital and College of Medicine, Taipei 100, Taiwan

## Abstract

**Background:**

MicroRNAs (miRNAs) are a class of endogenous, small and highly conserved noncoding RNAs that control gene expression either by degradation of target mRNAs or by inhibition of protein translation. They play important roles in cancer progression. A single miRNA can provoke a chain reaction and further affect protein interaction network (PIN). Therefore, we developed a novel integrative approach to identify the functional roles and the regulated PIN of oncomirs.

**Results:**

We integrated the expression profiles of miRNA and mRNA with the human PIN to reveal miRNA-regulated PIN in specific biological conditions. The potential functions of miRNAs were determined by functional enrichment analysis and the activities of miRNA-regulated PINs were evaluated by the co-expression of protein-protein interactions (PPIs). The function of a specific miRNA, miR-148a, was further examined by clinical data analysis and cell-based experiments. We uncovered several miRNA-regulated networks which were enriched with functions related to cancer progression. One miRNA, miR-148a, was identified and its function is to decrease tumor proliferation and metastasis through its regulated PIN. Furthermore, we found that miR-148a could reduce the invasiveness, migratory and adhesive activities of gastric tumor cells. Most importantly, elevated miR-148a level in gastric cancer tissues was strongly correlated with distant metastasis, organ and peritoneal invasion and reduced survival rate.

**Conclusions:**

This study provides a novel method to identify active oncomirs and their potential functions in gastric cancer progression. The present data suggest that miR-148a could be a potential prognostic biomarker of gastric cancer and function as a tumor suppressor through repressing the activity of its regulated PIN.

## Background

MicroRNAs are small non-coding, single stranded RNA of ~22 nucleotides in length that are abundantly found in eukaryotic cells [1]. The complementarity is between seed regions of mature miRNAs and their target messengers, enabling miRNA-mRNA interactions to occur. These interactions are crucial for post-transcriptional regulation of target gene expression by obstructing the mRNA translation or stability in the cytoplasm, and depend on both the expression levels of miRNAs and target mRNAs [2,3]. Some miRNAs are reported as oncomirs which could function as either oncogenes or tumor suppressors [4]. For example, miR-21 decreased tumor suppressor Pdcd4 expression and promoted invasion, intravasation and metastasis in colorectal cancer [5]. MiR-21 also regulated PTEN-dependent pathway and affected cell growth, migration and invasion of hepatocellular cancer [6]. Moreover, miR-21 and miR-155 were significantly associated with cancer metastasis and patients with higher miR-21 or miR-155 expression levels had worse survival [7]. MiR-155 was found to be up-regulated during innate immune response and autoimmune disorders as well as in various malignancies. In addition, miR-155 targeted tumor suppressor WEE1 homolog-S. pombe (WEE1) and caused gene alternation required for cancer development and progression [8]. On the other hand, let-7 decreased cell proliferation and migration of glioblastoma and reduced tumor size in xenograft model [9]. Let-7 prevented early cancer progression through suppressing embryonic gene high mobility group, A2 (HMGA2) expression [10]. Metastatic gastric cancer cells secreted let-7 via exosomes into the extracellular environment to maintain their oncogenesis [11].

Recently, many reports showed that they successfully identified miRNA targets using miRNA expression profiles [12,13]. Huang *et al*. used RNA expression data to identify 1597 high-confidence target predictions for 104 human miRNAs and further verified let-7b was down-regulated in retinoblastoma and CDC25A and BCL7A were targets of let-7b using qRT-PCR and microarray profile. Li *et al*. combined sequence complementarity, miRNA expression level, and protein abundance to identify miRNA targets for elevating their predictions. They also found that translational repression of targets by miRNAs was dominant mechanism in miRNA regulation. Moreover, sequence-based computational methods have been broadly used to predict putative miRNA targets [14], and can reach pretty good prediction rate, including cancer-related miRNAs [15,16]. Previous report has also indicated that computational prediction should take into account the expression profiles of both miRNA and mRNA [3]. Therefore, the development of an integrative approach that incorporated expression data to facilitate the identification of condition-specific targets of miRNAs becomes increasingly important.

MicroRNA can obstruct the translation of mRNA, thereby directly affecting protein abundance [15,16] and PINs [17,18]. For example, Yu *et al. *analyzed correlations between transcription factors (TFs) and miRNAs and further discovered that different regulatory networks formed by miRNA and TFs were involved in different biological functions [17]. Additionally, Liang *et al. *found global correlation between miRNA repression and protein-protein interactions and elucidated the related biological processes of miRNA-regulated PINs [18]. PINs are sets of interactions formed by two physically interacting proteins, which are fundamental to most biological processes [19]. With the accumulation of PPI data, it is becoming increasingly possible to understand the architecture and function of the cellular network by computational approaches [20,21]. Recently, we characterized the global properties of miRNA regulation in human PIN and proposed possible mechanisms of how these miRNAs regulate PINs [22]. Additionally, previous studies have demonstrated that miRNAs can affect specific biological functions which are involved in tumorigenesis and cancer progression through the regulation of a small number of genes within biological networks, such as PINs [23,24]. Thus, assessing how miRNAs affect PINs could facilitate the discovery of potential miRNA-related networks and allow the characterization of associated biological functions.

**Figure 1:**
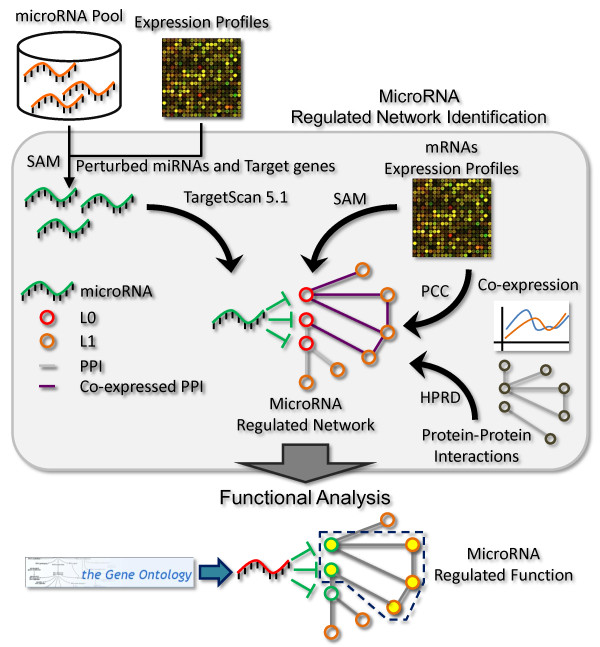
**Expression profiles of miRNAs and genes were used to discover condition-specific targets of miRNAs**. The miRNA-regulated PINs were denoted as the PIN which is formed by their differentially expressed targets (L0) and interacting partners (L1). The enrichment of CePPIs involved in the miRNA-regulated PIN was utilized to evaluate the activity of the network. The potential miRNA-regulated functions were predicted by functional enrichment analysis.

In this study, we proposed an integrative analysis which suggested that miRNA-regulated PINs could be identified based on the combination of down-regulated miRNAs and up-regulated mRNAs. The procedure for miRNA-regulated PIN identification and analysis is illustrated in Figure 1. We subsequently showed that the networks that were modulated by these down-regulated miRNAs in tumors tended to be activated in gastric cancer. Among these was the miR-148a-regulated PIN, which was involved in metastasis-related biological processes that were associated with tumor suppression. In particular, miR-148a was shown to inhibit tumor invasion, migration, adhesion and cell growth, and prolonged patient survival. These findings suggest that miR-148a is not only a potential prognostic marker that can be used for the detection of human gastric cancer, but it can also suppress gastric cancer progression through the regulation of its associated network.

## Results

### MicroRNA-regulated PINs in Gastric Cancer

Although microRNAs are known to reduce the levels of their target mRNAs [25], expression of a miRNA target may not always co-vary, in the reverse direction, with its miRNA regulator, due to the presence of primary TF regulation. A reverse correlation in expression profiles between a miRNA and corresponding predicted targets increases the confidence of the conditional miRNA-target interaction (See Additional file 1). Based on these observations, we combined differentially expressed miRNAs and target genes, down-regulated (up-regulated) miRNAs versus up-regulated (down-regulated) targets, to identify the miRNA-regulated PINs in gastric cancer. The miRNA-regulated PINs were established according to the proteins encoded by differentially expressed target genes and their interacting partners in the human PIN. However, only 23 significantly down-regulated miRNAs and no significantly up-regulated miRNAs were found (see Methods in Additional file 1). Consequently, we identified 23 PINs that were modulated by 23 down-regulated miRNAs in gastric cancer. Furthermore, the activities of miRNA-regulated PINs in normal and tumor tissues, activated or inactivated, were investigated on the basis of the enrichment of co-expressed PPIs (CePPIs) in the network (Additional file 1, Table S1, S2). Among the 23 down-regulated miRNA-regulated PINs, 70% (16/23) of them were activated in tumors (Fisher's exact test, *P *< 0.001). On the other hand, 51% (20/39) of the remaining 39 unchanged miRNA-regulated PINs were inactivated in both tumor and normal tissue (Fisher's exact test, *P *= 0.003). Considering that HPRD PIN was incomplete, we randomly removed some proportions of PPIs, from 5% (1744 PPIs) to 20% (6974 PPIs), and evaluated the effects on the activities of miRNA-regulated networks. Most of the miRNAs remained activated in tumor (~75%) with 1000 repeated random removal tests (Additional file 1, Figure S1), indicating that the results were robust against missing PPIs. Thus, we conclude that 16/23 networks are activated in gastric cancer and that these networks are modulated as a result of down-regulated miRNAs expression. Therefore, we suggest that the 16 down-regulated miRNAs act as oncomirs and function as tumor suppressors.

Additionally, we assessed the discrimination power of miRNA expression levels to classify normal and tumor samples by calculating the receiver operating characteristic (ROC) curves and the area under the curve (AUC). Among the 23 down-regulated miRNAs, the one that provided the best discrimination was miR-29c (AUC = 0.831, *P *< 0.001), which gave an overall correct classification of 77%. Moreover, of the 16 oncomirs, miR-29c, miR-768-3p, miR-26a, miR-143 and miR-148a were found to give an AUC of more than 0.7 (*P *< 0.05) individually. When all the 16 oncomirs were combined, the overall correct classification was elevated to 93% (AUC = 0.981, *P *< 0.0001). Among the remaining 7 down-regulated miRNAs, only miR-16 and miR-145 were found to have an AUC of more than 0.7. When these 7 miRNAs combined, the overall correct classification was 82% (AUC = 0.888, *P *< 0.0001) (Additional file 1, Table S3). Taken together, the 16 oncomirs showed greater discrimination between tumor and normal tissues than the remaining 7 down-regulated miRNAs.

We also investigated the relationship between the expression levels of the 23 down-regulated miRNAs and survival rates. The median was used as a cut-off value and as a result, the expression levels for each miRNA were divided into two groups; high and low expressed. The maximum of the difference in survival rates (D_max_) between high and low groups was further calculated (Additional file 1, Figure S2A). Our findings indicated that the D_max _among the 16 oncomirs was more significant (*P *< 0.0001, Additional file 1, Figure S2B) than that in the remaining 7 down-regulated miRNAs (paired Wilcoxon rank sum test, *P *= 0.016, Additional file 1, Figure S2C). These results suggest that the 16 oncomirs may act as better prognostic markers for gastric cancer in comparison to the remaining 7 down-regulated miRNAs. Therefore, we conclude that the 16 oncomirs have the potential to suppress tumor biogenesis.

### The Potential Functions of Oncomir-regulated PINs

A key question is whether the 16 oncomirs regulate tumor progression-related biological processes in gastric cancer. To address this, we developed a PIN-based approach to reveal the possible functional roles of miRNAs (see Methods in Additional file 1). Our findings showed that union of miRNA-regulated targets (L0) and their interacting partners (L1) displayed the most enriched GO functional terms and trees on average (Additional File 1, Table S4). The low coverage of L0 genes (<50%) (Additional File 1, Table S4), indicated that the functional relationship between L0 genes was weak. However, the high coverage of union genes (approximately 100%) (Additional File 1, Table S4) suggested that there was a very strong functional relationship between these genes. Additionally, our previous analysis on miRNA regulation in human PIN [22] also suggested that miRNA-regulated genes and their interacting partners jointly showed significantly higher modularity than miRNA-regulated genes alone. Based on these results, analysis of regulatory mechanisms of miRNAs in the context of the PIN enables a more accurate prediction of the potential functions of miRNAs.

Applying this PIN-based approach to 16 oncomirs, the enriched biological processes of their regulated PINs can be discovered (Additional File 1, Table S5). It was observed that most of the enriched biological processes were related to tumor progression. For instance, miR-142-3p- and miR-768-3p-regulated PINs were related to apoptosis and cell cycling, respectively. Interestingly, the miR-148a-regulated PIN was the only network that was associated with cancer metastasis-related functions, such as integrin-mediated signaling, cell-matrix adhesion and wound healing. Therefore, miR-148a was chosen for the further studies.

### MiR-148a-regulated PIN and Its Potential Functions in Gastric Cancer

**Figure 2:**
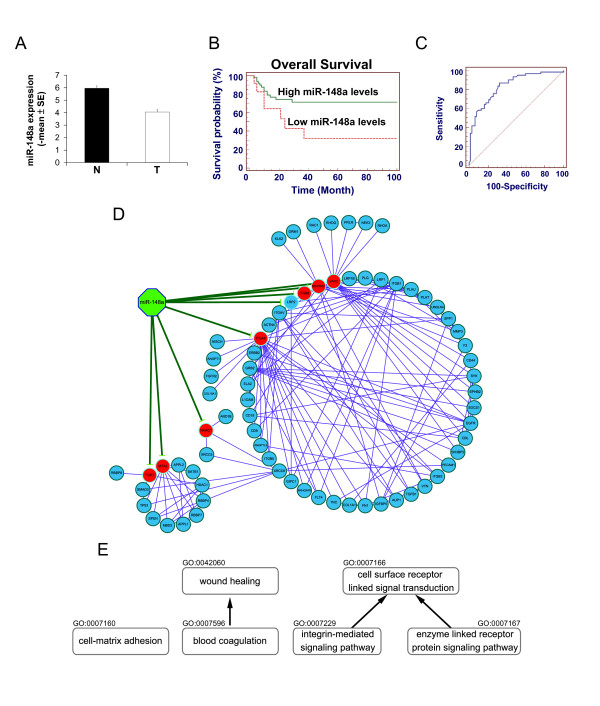
**miR-148a and its regulated PIN**. (A) miR-148a expression levels (Mean ± standard error) were detected and normalized to U6 snRNA by qRT-PCR (*P *< 0.0001, paired *t*-test). (B) The effect of miR-148a expression levels on patient survival was measured. Cut-off value of 0.101 was determined based on the finding that miR-148a expression levels in 60% of patients (37 out of 62) that survived for a period of 2 to 140 months after surgical resection were above 0.101. Solid and dotted lines represent high (≧0.101, N = 50) and low (<0.101, N = 12) miR-148a levels. (C) The AUC of miR-148a was 0.84 (0.76-0.90, 95% confidence interval) (ROC curves analysis, *P *= 0.0001). (D) Red nodes represent up-regulated target genes and blue nodes represent their interacting partners within the human PIN. Green edges represent predicted interactions between miRNAs and targets and purple edges represent PPIs between proteins. (E) Nodes represent enriched GO terms in the miR-148a-regulated PIN and edges represent the relationships in the GO. *P*-value of each enriched GO terms: cell-matrix adhesion: 9.5619E-6, wound healing: 5.8018E-5, blood coagulation: 8.0094E-4, cell surface receptor linked signal transduction: 5.3054E-5, integrin-mediated signaling pathway: 3.1108E-6, enzyme linked receptor protein signaling pathway: 2.3001E-4.

To further confirm the abundance of miR-148a in tumor tissues, we performed qRT-PCR in 62 paired tumor and normal tissues and found its expression levels in tumor tissues were significantly lower than those in normal tissues (*P *< 0.0001, paired *t*-test) (Figure 2A), which was consistent with our miRNA microarray data. Additionally, the impact of miR-148a expression levels on the prognosis of gastric cancer patients by Kaplan-Meier survival analyses was studied in the 62 paired tissues. Patients with high miR-148a expression levels showed significantly higher 5-year overall survival rates (71.4%, log-rank test, *P *= 0.03; Figure 2B) compared with patients with low miR-148a levels (32.1%, log-rank test, *P *= 0.03). Univariate analysis showed that miR-148a and early stages correlated with better survival, whereas peritoneal and vascular invasion predicted very poor outcomes. On multivariate analysis, miR-148a retained an independent prognostic power on overall survival (HR = 1.69; *P *= 0.002) (Additional File 1, Table S6). A ROC curve was also used to evaluate miR-148a as a diagnostic marker for gastric cancer. The AUC was then used as an indicator of the capacity of miR-148a to act as a diagnostic marker, with higher AUC values reflecting a higher diagnostic potential. ROC curve analysis of miR-148a showed that it had an AUC of 0.84 (ROC curves analysis, *P *= 0.0001, Figure 2C). These results indicate that miR-148a could discriminate between normal and tumor tissues and serve as an effective prognostic marker for gastric cancer. Based on these results, we conclude that miR-148a is highly associated with gastric cancer. The miR-148a-regulated PIN was visualized in Figure 2D and the enriched biological processes of this network are shown in Figure 2E. The significantly over-represented GO functional terms were separated into three functional groups, including cell-matrix adhesion (hypergeometric test: *P *= 9.5619E-6), wound healing (*P *= 5.8018E-5) and cell surface receptor linked signal transduction (*P *= 5.3054E-5). These three enriched GO functions suggest that miR-148a is related to cancer metastasis and is likely to regulate these three functions through its regulated PIN.

### PAI-1, ITGB8, VAV2 and ITGA5 Are Oncogenes and Direct Targets of MiR-148a

**Figure 3:**
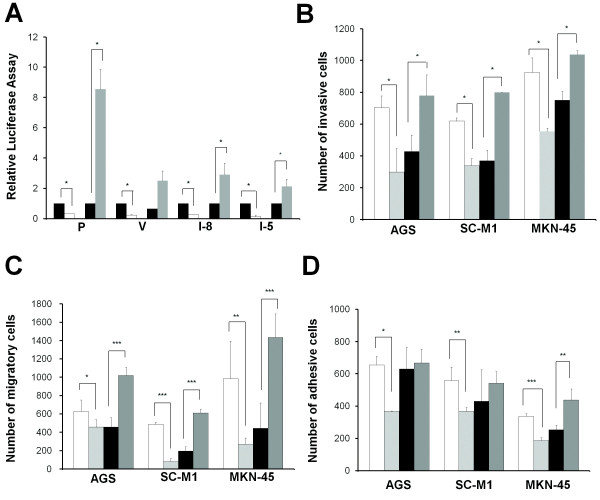
**The relationship between miR-148a and PAI-1 (P), VAV2 (V), ITGB8 (I-8) and ITGA5 (I-5)**. (A) White and light gray bars represent miR-148a precursor and inhibitor, respectively. Black and dark gray represent precursor and inhibitor negative controls, respectively. Over-expressed miR-148a reduces invasion (B), migration (C) and adhesion (D) of tumor cells. Invasive, migratory and adhesive activities of tumor cells were measured after transfection for 48hr with miR-148a precursor (light gray bar) and inhibitor-treated (dark gray bar) AGS, SC-M1 and MKN-45 cell lines. Precursor (white bar) and inhibitor (black bar) negative controls were used (*t*-test, *P < 0.05; **P < 0.01; ***P < 0.001).

Four target genes of miR-148a were identified to be up-regulated within its regulated PIN, including PAI-1 (a coagulation factor), ITGB8 (an adhesion factor), VAV2 and ITGA5 (involved in the integrin-mediated signaling pathway). To further explore whether miR-148a affected cancer metastasis through its regulated PIN, a luciferase reporter assay was performed to analyze the relationship between miR-148a and these target genes. miR-148a over-expression significantly reduced the expression levels of these target genes, while their expression levels were significantly elevated in anti-miR-148a inhibitor-transfected tumor cells (*t*-test, *P *< 0.05, Figure 3A). We carried out luciferase assay for site mutant to further ensure the correlations between miR-148a and these four genes (Additional file 1, Figure S3). We constructed each putative miR-148a target sites or its site mutation in sequences corresponded to seed sequence of miR-148a into a pMIR-REPORT luciferase expression vector (Additional file 1, Figure S3A) and analyzed reporter assays (Additional file 1, Figure S3B-S3E). Our results showed that the repressions by miR-148a in these four genes were completely abolished. These results suggest that these four genes are direct targets of miR-148a. These observations suggest that miR-148a plays a significant role in affecting the biological functions of gastric cancer cells by regulating the expression of target genes within its regulated PIN.

In addition to being markers for the detection and prognosis of many types of cancers [26-35], these four genes have previously been reported to be implicated in the promotion of cell invasion, migration, adhesion, growth and angiogenesis, which suggests a possible role for these genes in the regulation of tumor oncogenesis and progression. To determine whether these genes played a role in the oncogenesis of gastric cancer, their expression levels in tumor tissues were measured by immunoblotting. All four genes showed higher expression levels in tumor tissues compared with normal tissues (Additional file 1, Figure S4), indicating that they might have potential oncogenic functions and were likely to be key downstream effectors of miR-148a in this network.

### The Correlation Between miR-148a and Clinicopathological Factors

To evaluate the clinical significance of miR-148a in gastric cancer, the relationship between miR-148a expression levels in tumor tissues and the degree of metastasis was analyzed (see Methods in Additional file 1). Clinical analyses revealed that high expression levels of miR-148a significantly correlated with a reduction in distant metastasis (*t*-test, *P *= 0.043), organ invasion (*t*-test, *P *= 0.013) and peritoneal invasion (*t*-test, *P *= 0.04) (Table 1). The relationship between miR-148a levels and all clinical factors was shown in Additional file 1, Table S7. These results suggest that miR-148a reduces the aggressiveness of gastric cancer. Interestingly, our findings revealed that several functions, including migration (integrin-mediated signaling pathway) and adhesion, were identified within the miR-148a-regulated PIN and were also associated with an aggressive tumor phenotype. This indicates that miR-148a likely plays an important role in regulating the malignant progression of tumor cells.

**Table 1:** The relationship between miR-148a expression levels and clinical factors.

	No. of Patients	%	Mean (2-ΔΔCt)	*p*-value
**Distant metastasis**^a^				0.043*
Negative	58	94	0.549	
Positive	4	6	0.238	
**Organ invasion**^a^				0.013*
Negative	27	44	0.630	
Positive	35	56	0.415	
**Peritoneal invasion**^a^				0.040*
Negative	17	27	0.676	
Positive	45	73	0.467	

T: tumor; N: normal. ^a ^*t*-test analysis. ^b ^One-way ANOVA analysis. *P < 0.05.

### MiR-148a Inhibits Cell Invasion, Migration, Adhesion and Growth

**Figure 4:**
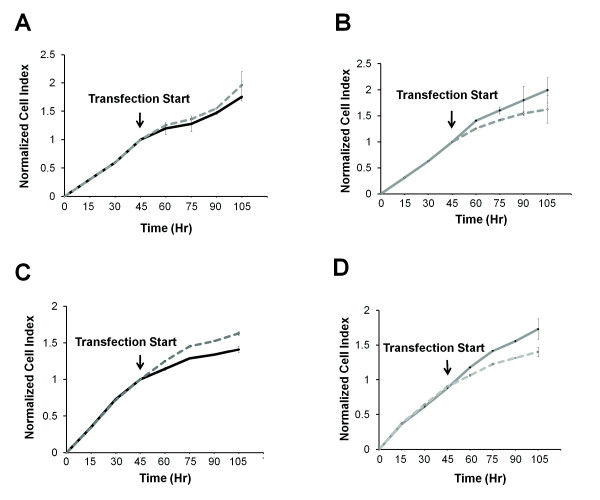
**Over-expressed miR-148a reduces cells growth**. Cell growth was monitored in real-time using the xCELLigence system. (A, B) AGS and (C, D) SC-M1 tumor cells were transfected with either miR-148a precursor or inhibitor and compared to their respective negative controls. The results indicated that miR-148a reduced tumor cell growth (*t*-test, *P *= 0.23, A and *P *< 0.001, C), while miR-148a inhibitor-transfected tumor cells showed increased cell growth (*t*-test, *P *< 0.001, B and *P *< 0.001, D). Black and gray solid lines represent miR-148a precursor and inhibitor, respectively. Dashed lines represent their negative controls. The *p*-value was calculated using a paired *t*-test.

*In vitro *invasion assays were performed to examine whether miR-148a suppressed more aggressive forms of tumors. Human gastric cancer AGS, SC-M1 and MKN-45 cell lines were transfected with a miR-148a precursor or an anti-miR-148a inhibitor. The results showed that over-expression of miR-148a in these cell lines significantly reduced tumor cell invasion, while anti-miR-148a-treated tumor cells showed elevated tumor cell invasion (Figure 3B). To determine whether miR-148a over-expression also affected tumor progression, migration, adhesion and proliferation assays were performed on miR-148a-transfected tumor cells. Our findings indicated that over-expressed miR-148a significantly reduced tumor cell migration and adhesion, while anti-miR-148a-treated tumor cells showed significantly elevated migratory and adhesive abilities (Figure 3C and 3D). Additionally, tumor cell growth was observed to be reduced in response to miR-148a over-expression (Figure 4A and 4C), but elevated in response to anti-miR-148a inhibitor treatment (Figure 4B and 4D). Taken together, these observations indicate that miR-148a can inhibit cell invasion, migration, adhesion and growth, thereby acting as a potent regulator of tumor suppression.

## Discussion

MiRNAs are known to function as gene silencers and their expression profiles have been reported to be negatively correlated with those of their target genes [25]. In particular, up-regulated target genes were found to be specific targets of down-regulated miRNAs in gastric cancer. In order to reveal the possible roles that these miRNAs play in gastric cancer, the predicted target genes of miRNAs were analyzed within the human PIN and it was shown that a stronger functional correlation existed for miRNA targets that were linked to specific PPI partners than for miRNA targets alone. Thus, this method provides us to understand the biological functions of miRNAs.

To find out the miRNA-regulated PINs in gastric cancer, the expression profiles of miRNAs were integrated and analyzed within the human PIN. Twenty three down-regulated miRNA-regulated PINs were identified based on their up-regulated target genes in gastric cancer. Among these, sixteen PINs were activated. The results suggest that repressing these miRNAs in gastric cancer can activate their regulated PINs. This may be due to miRNA-mediated regulation of pivot genes, such as hubs [22], or differentially expressed genes in the biological networks. Therefore, we suggest that these 16 down-regulated miRNAs act as oncomirs and function as tumor suppressors. Additionally, these 16 oncomirs were associated with increased tumor suppression potential and increased survival rates compared with the 7 remaining down-regulated miRNAs, suggesting that these oncomirs can be effective markers for the diagnosis and prognosis of gastric cancer.

Among 16 down-regulated miRNAs, we found that the miR-148a is down-regulated in gastric cancer and its regulated PIN was associated with tumor metastasis-related functions, such as integrin-mediated signaling, cell-matrix adhesion, wound healing and blood coagulation. These findings were validated by over-expressing miR-148a in AGS, SC-M1 and MKN-45 gastric cancer cell lines. While miR-148a over-expression led to a significant reduction in the invasive, migratory, adhesiveness and growth properties of gastric cancer cells, miR-148a inhibitor-treated tumor cells enhanced these effects. Lujambio *et al. *found miR-148a inhibited metastasis formation in xenograft models [36] and Chen *et al. *showed that expression level of miR-148a in human gastric cancer was significantly lower than that in their matched nontumor adjacent tissues [37]. These results support our findings, miR-148a regulates malignant progression in gastric cancer. Additionally, patients with high miR-148a expression levels exhibited a significantly better overall survival rate than those with low miR-148a levels, suggesting that miR-148a might function as a tumor suppressor and could potentially serve as a diagnostic and prognostic marker for gastric cancer.

We next ascertained whether miR-148a-mediated down-regulation of PAI-1, VAV2, ITGA5, and ITGB8 expression resulted in the inhibition of malignant progression of tumor cells. These genes were identified as up-regulated targets of miR-148a within its regulated PIN and have been reported to have a high oncogenic potential and are associated with aggressive tumor cell phenotypes [26-33]. Herein, we showed that their expression levels were reduced in response to miR-148a over-expression but elevated in response to anti-miR-148a inhibitor treatment. Overall, these results suggest that miR-148a influences the tumor progression-related biological functions of cancer cells by regulating a small subset of cancer-relevant genes within its regulated PIN.

## Conclusions

In conclusion, a novel integrative network-based approach was used to demonstrate the dynamic and conditional nature of miRNA-regulated gene expression and to discover the potential functions of these miRNAs in gastric cancer. Furthermore, this approach facilitated the identification of 16 activated oncomir-regulated PINs which might be used as possible diagnostic and prognostic markers of gastric cancer. In particular, miR-148a was identified as a potential prognostic marker in gastric cancer patients, with the ability to function as a tumor suppressor through its regulated PIN. This study not only provides an insight into the miRNA-regulated PINs that are involved in the pathogenesis of gastric cancer; it also shows that a network-based approach can be used to identify novel diagnostic and prognostic markers of disease.

## Methods

### Expression Profiles of MiRNAs and mRNAs

The mRNA expression profiles of gastric cancer were retrieved from Gene Expression Omnibus (GEO), accession number GSE13911 [38]. The miRNA expression profiles were obtained from 22 paired tumor and non-tumor specimens of gastric cancer patients who underwent curative gastrectomy at National Taiwan University Hospital (Taipei, Taiwan) between 2001 and 2006. Gastric cancer and normal tissues from these patients were collected at the National Taiwan University Hospital after receiving patient written informed consent. The protocol was approved by the local ethics committees, National Taiwan University Hospital. We have submitted the miRNA microarray data to the GEO database and the series record is GSE28700. All the human tissue samples have been approved and human subject confidentiality has been protected by the Institute Review Board (IRB, 9261700703). The characteristics of these tissue specimens are described in Methods in Additional file 1.

### MiRNA-regulated PIN Identification and Analysis

The procedure for miRNA-regulated PIN identification and analysis is illustrated in Figure 1. For identifying the miRNA-regulated PINs, significantly differentially expressed miRNAs and genes were determined by Significance Analysis of Microarrays (SAM) [39] (delta of 5 and fold change of 2), implemented in MultiExperiment Viewer (MeV) v4.5.1 [40]. As we applied this cutoff, we could control the false discovery rate was less than 0.00001% (evaluated by SAM). Finally, only 23 significantly down-regulated miRNAs and no significantly up-regulated miRNAs were found. The regulated network of each miRNA consists of its significantly up/down-regulated target genes and their interacting partners in the human PIN. The putative target genes of miRNAs were obtained from TargetScan 5.1 [41], and the human PIN was from Human Protein Reference Database (HPRD) [42]. The enrichment of co-expressed PPIs (CePPIs) involved in the network was used to evaluate the activation state of miRNA-regulated PINs in tumor and normal tissues (see Methods in Additional File 1). MicroRNA-regulated biological functions were predicted by functional enrichment analyses of their regulated PINs (see Methods in Additional File 1).

### Cell Culture and Authentication of Cell Lines

Human gastric cancer AGS, SC-M1 and MKN-45 cells were obtained from the cell line databank in National Taiwan University Hospital in 2008 and have been tested and authenticated in our laboratory on a monthly basis. These cells were last tested by morphology check using microscope and mycoplasma detection using Hoechst 33258 in March 2010. These cells were maintained in RPMI-1640 medium supplemented with 10% fetal bovine serum (FBS; Invitrogen, Carlsbad, California, U.S.A) and cultured at 37°C in an atmosphere of 5% CO_2_.

### qRT-PCR for MiRNA Expression

Total RNA from 62 paired tumor and normal tissues was obtained using Trizol (Invitrogen) and the mirVana miRNA isolation kit (Applied Biosystems, California, U.S.A.) for miRNA detection.

### Invasion Assays Using Boyden Chamber Assays

Cells were transfected with a miR-148a precursor and a miR-148a inhibitor. After 24 hr, invasion assays were performed with modified Boyden chambers containing filter inserts (pore size, 8 μm) coated with Matrigel (35 μg, BD Biosciences) based on previously described methods [43].

### Wound Healing Assay

Cell monolayers were wounded after transfection for 48 hr by scratching with a 200 μl pipette tip. Debris was removed by washing and the scratched cells were incubated for 24 hr. Distances between wound edges were measured at five different locations under × 20 magnification by a microscope and analyzed using Metamorph version 7.0 software.

### Cell Adhesion Assay

Adhesion assays were performed using Matrigel coated 96-well plates that were incubated at 37°C for 1 hr. Details are described in Methods in Additional File 1.

### Cell Proliferation Assay

Cell growth was measured in real-time using the xCELLigence system (Roche Applied Science and ACEA Biosciences). 7.5 × 10^3 ^cells were seeded in each well of an E-plate 16 (Roche Applied Science and ACEA Biosciences) and incubated at 37°C for 48 hr. After incubation, cells were transfected with a miR-148a precursor and inhibitor and the growth of living cells were monitored in real-time.

Additional information regarding details of individual experimental procedures can be found in the Additional files submitted along with the main manuscript.

## Competing interests

The authors declare that they have no competing interests.

## Authors' contributions

CWT, CCL, HCH, and HFJ carried out the analysis, and drafted the manuscript. CWT carried out biological experiments. CCL implemented the computational method. CNC undertook clinical data analysis. HCH, CNC and HFJ conceived and directed the project, participated in the design and coordination of the study, and edited the manuscript. All authors read and approved the final manuscript.

## Supplementary Material

Additional file 1 **figures, tables, and methods**. This file contains Figures S1-S9, Tables S1-S7. It includes the analyses of clinical data of oncomirs, enriched functions of oncomir-regulated PIN, the expression of miR-148a targets in tumor tissues, the relationship between miR-148a and clinical factors, and the detailed methods. Click here for file
