# Supplementary material for: Integrative network analysis reveals active microRNAs and their functions in gastric cancer
Source: BMC Syst Biol. 2011 Jun 26;5:99. doi: 10.1186/1752-0509-5-99 (PMC3142228; doi:10.1186/1752-0509-5-99)
Supplement: Additional file 1 — figures, tables, and methods. This file contains Figures S1-S9, Tables S1-S7. It includes the analyses of clinical data of oncomirs, enriched functions of oncomir-regulated PIN, the expression of miR-148a targets in tumor tissues, the relationship between miR-148a and clinical factors, and the detailed methods. [file 1752-0509-5-99-S1.DOC]

**Integrative network analysis reveals active microRNAs and their functions in gastric cancer**

Chien-Wei Tseng, Chen-Ching Lin, Chiung-Nien Chen, Hsuan-Cheng Huang, Hsueh-Fen Juan

**Additional File**

**Figures:** Figure S1 – S9

**Tables:** Table S1 – S7

**Methods**

**References**

**Figures**

**
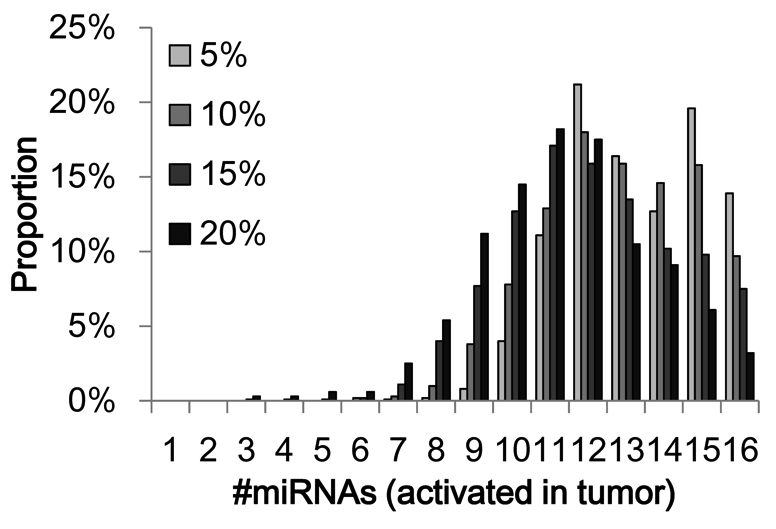
**

**Figure S1. The robustness of 16 tumor-activated miRNA-regulated PINs to incomplete human PIN.** From 5% (1744 PPIs) to 20% (6974 PPIs) PPI removing ratio, most of miRNA-regulated PINs were activated in tumor (about 75%, 12 / 16).

**
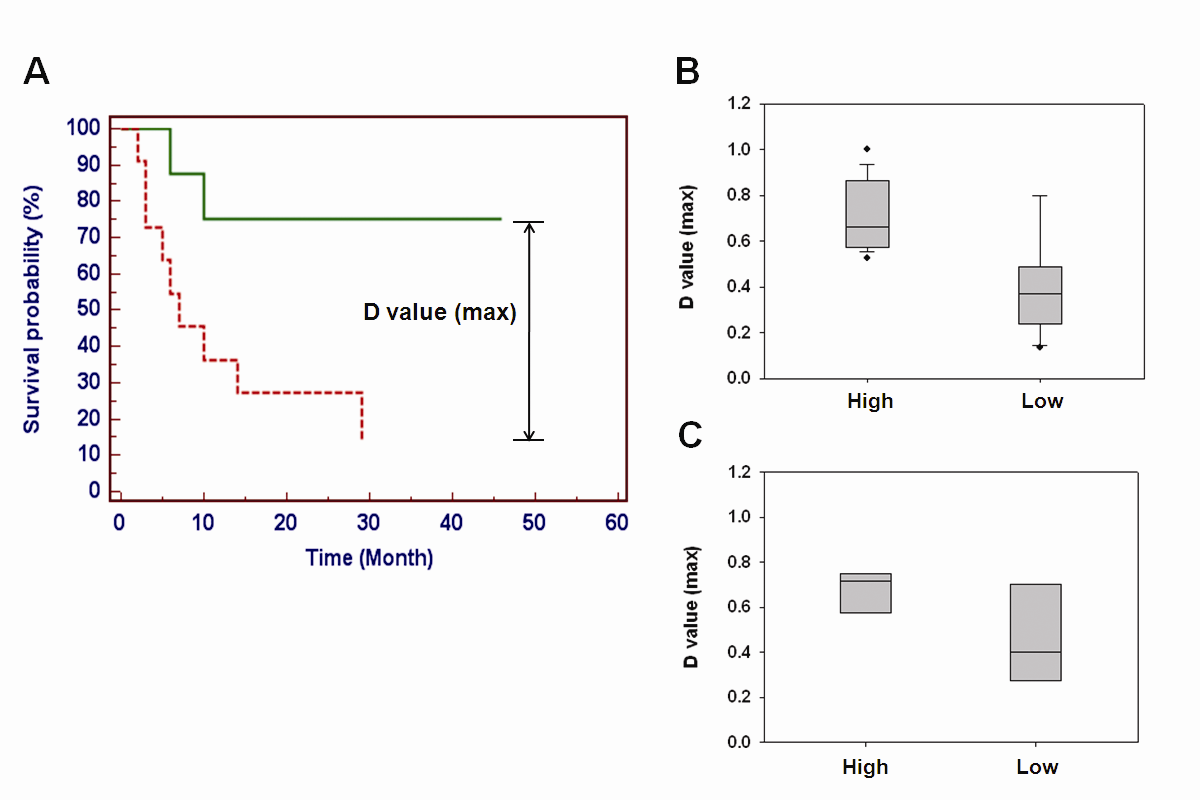
**

**Figure S2. Kaplan-Meier survival curves of 23 downregulated miRNAs in gastric cancer.** (A) The median of 22 paired miRNA expression values that was obtained by miRNA microarray was defined as the cutoff value. The green line represents high miRNA expressing patients and the red line represents low miRNA expressing patients. The D value was calculated by analyzing the maximum difference in survival rate between high and low groups. The difference in D values between high and low groups for the 16 oncomir in Table S1 was more significant (*P* < 0.0001, B) than in the 7 remaining down-regulated miRNAs (*P* = 0.016, C) (paired Wilcoxon rank sum test).


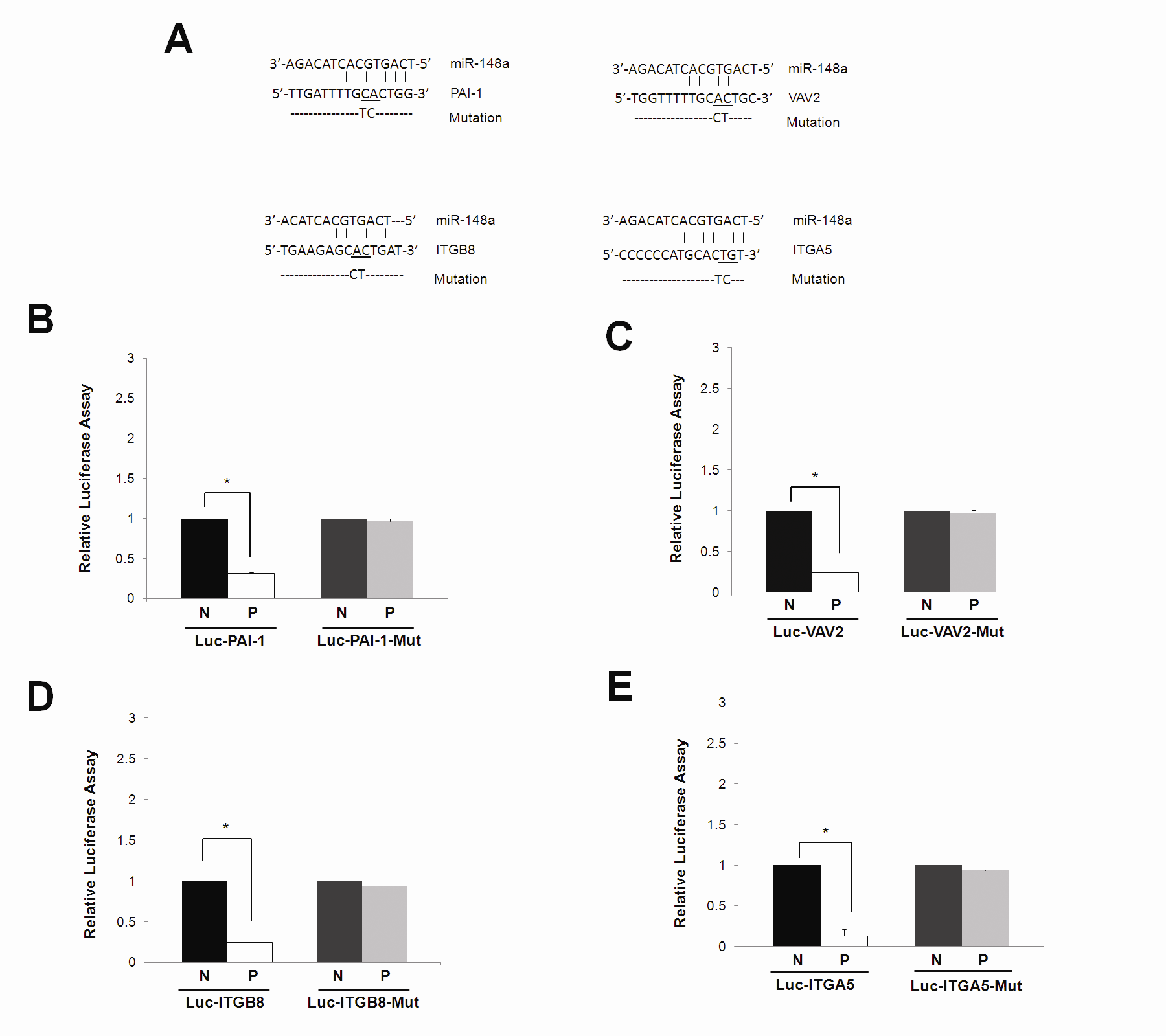

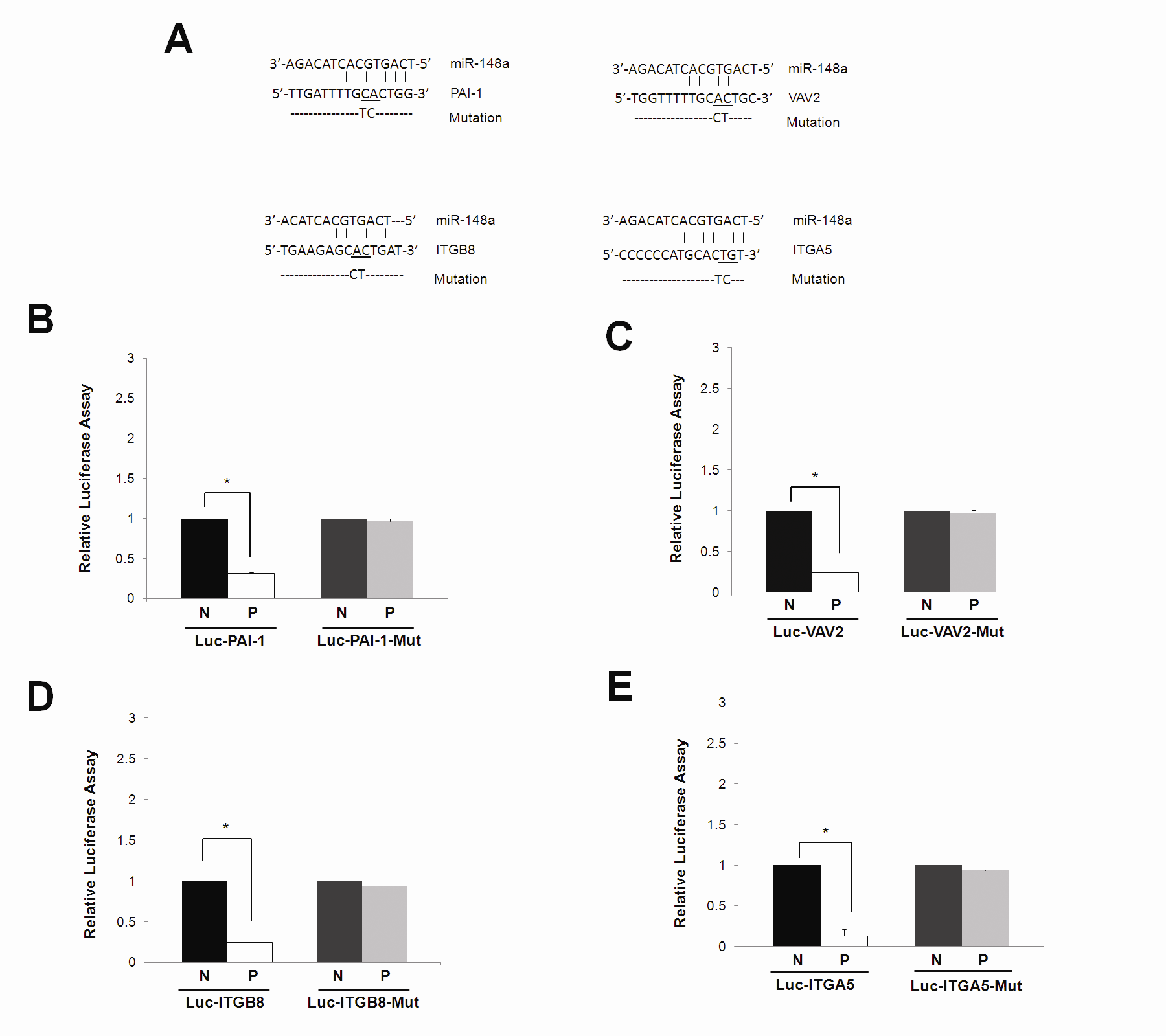


**Figure S3. These genes are direct targets of miR-148a.** (A) Schematic diagram of miR-148a-target sites and sites mutation in PAI-1, VAV2, ITGB8 and ITGA5. (B-E) Luc-PAI-1, Luc-VAV2, Luc-ITGB8 and Luc-UTGA5 represented AGS cells were transfected with pMIR-REPORT luciferase expression vector containing each putative miR-148a target sites. Mut represented their sites mutation in sequences. P and N represented miR-148a precursor and negative control, respectively (**P* < 0.05).


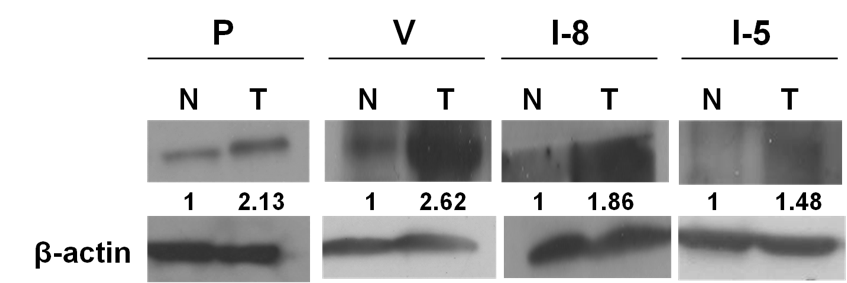


**Figure S4. The expression levels of PAI-1 (P, 50kDa), VAV2 (V, 95kDa), ITGB8 (I-8, 85kDa) and ITGA5 (I-5, 150kDa) in paired tumor (T) and normal (N) tissues were measured by immunoblotting.** β-actin was used for normalization.


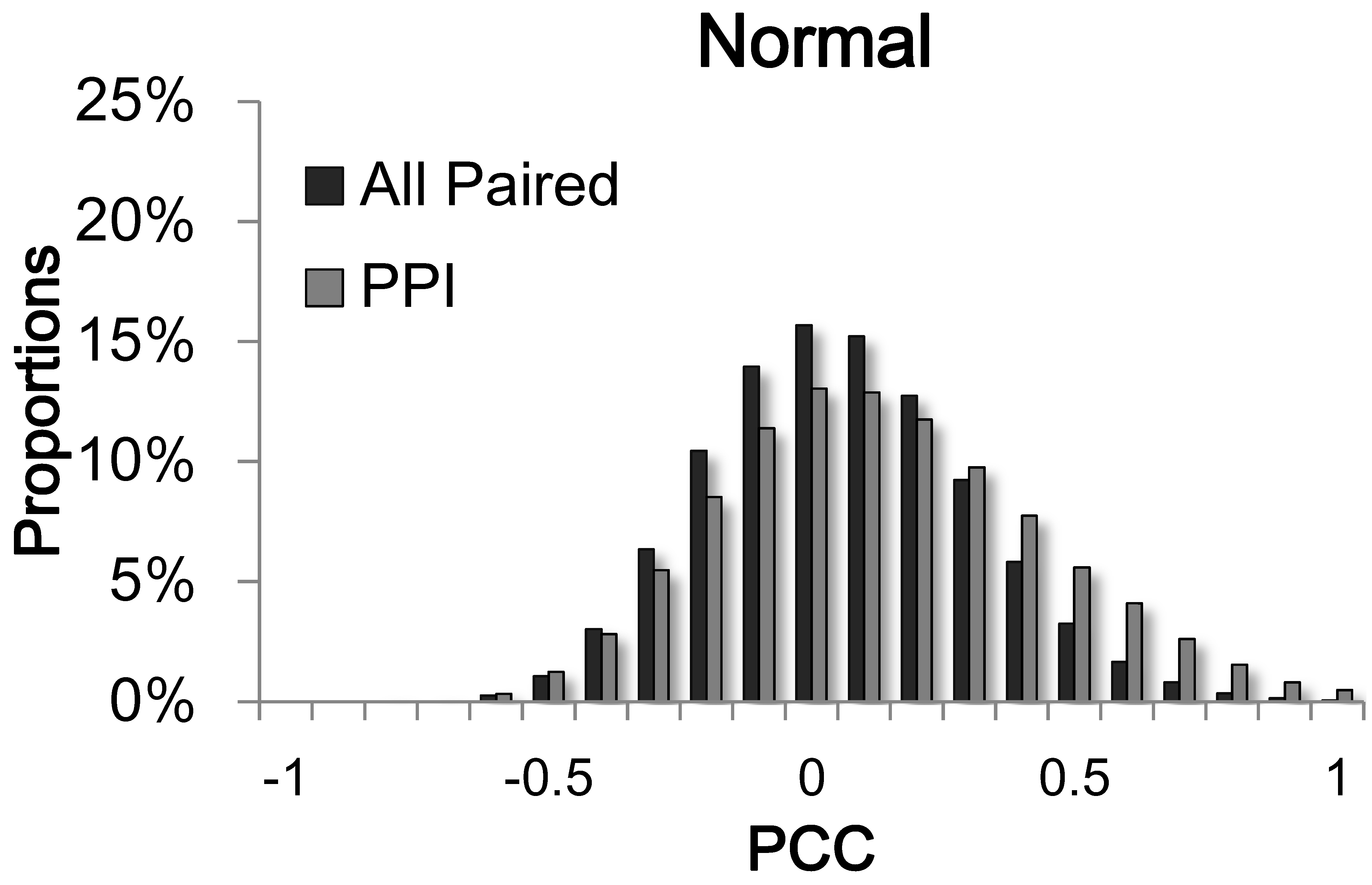


0.3


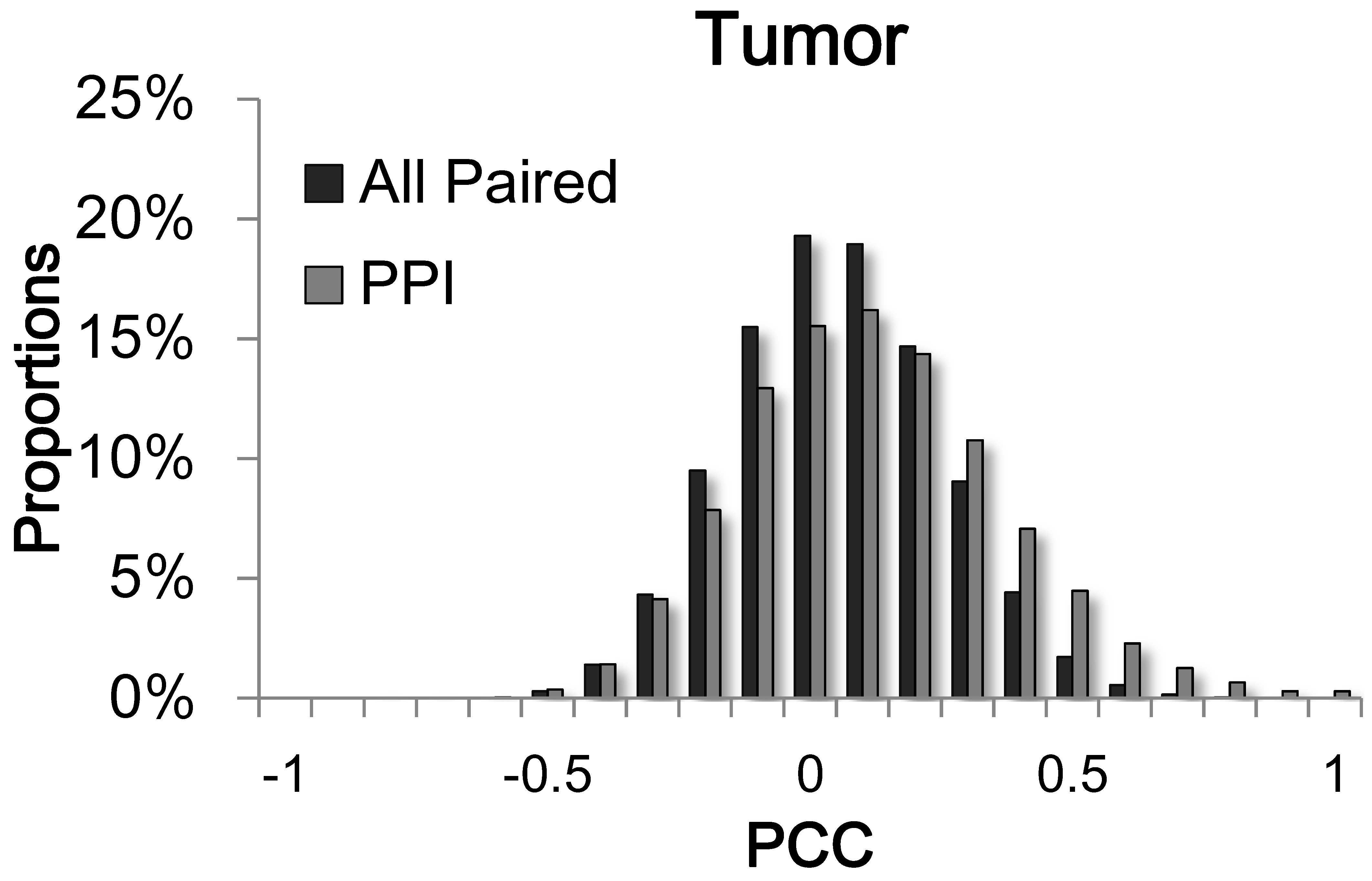


0.3

**Figure S5. Distributions of PCCs between PPI pairs and all gene pairs in normal and tumor samples.** If PCC > 0.3, the proportion of PPI pairs is higher than that of all gene pairs.


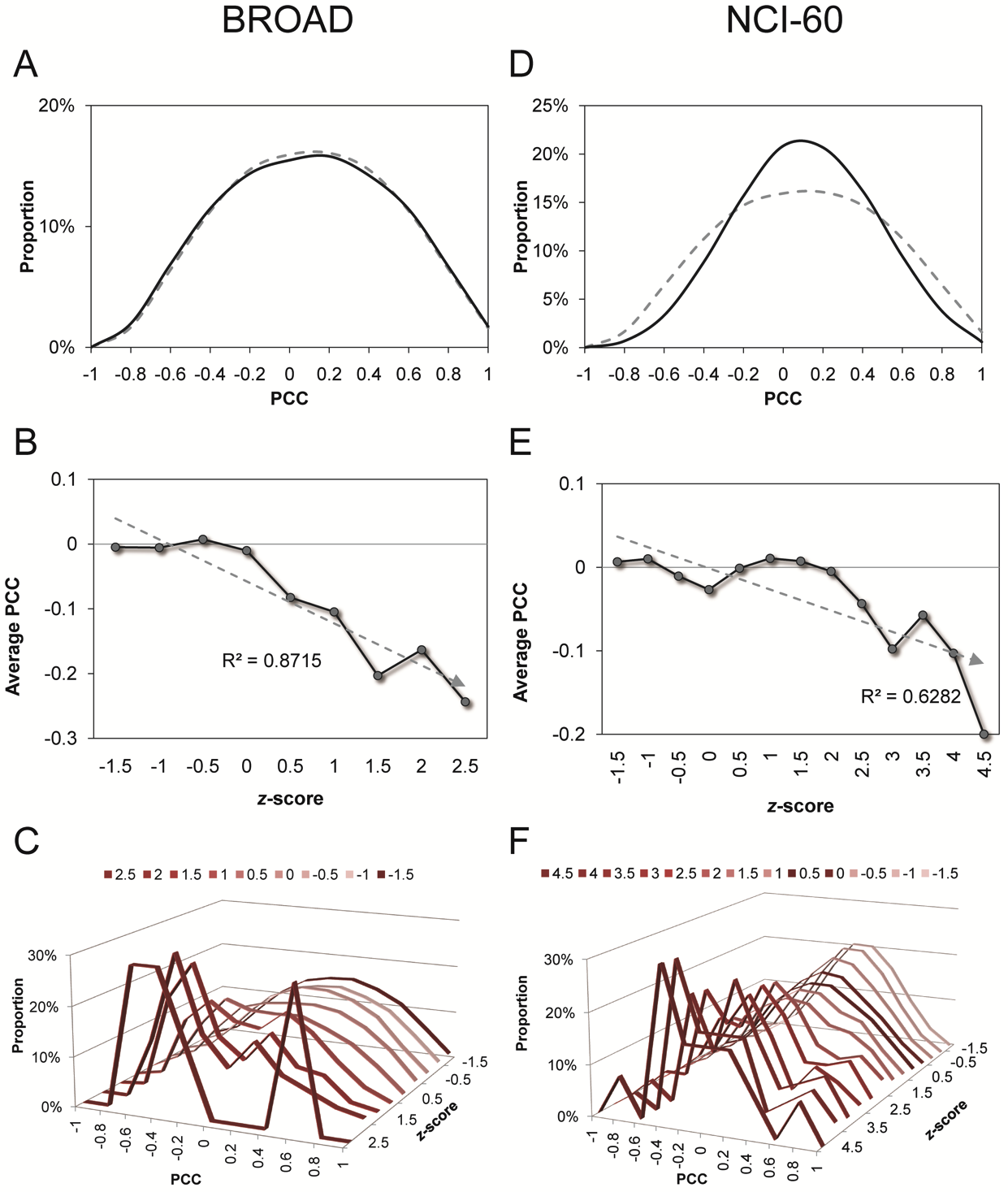


**Figure S6. The Pearson correlation coefficient (PCC) between expression profiles of miRNAs and mRNAs of the NCI-60 and BROAD dataset.** (A) (D) Solid line: the distribution of PCC between the expression profiles of miRNAs and corresponding predicted targets; dashed line: the distribution of PCC between the expression profiles of miRNAs and non-targets.


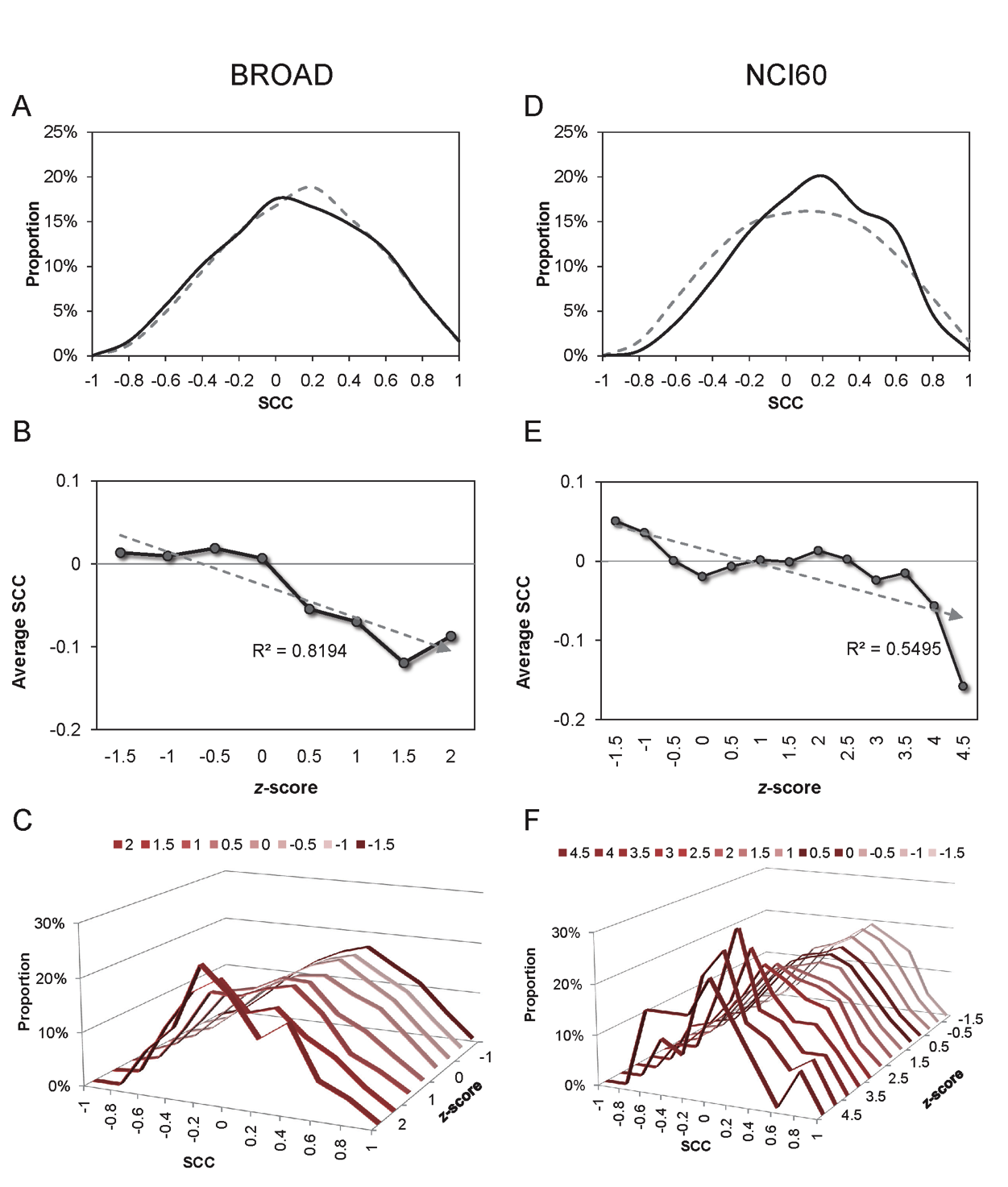


**Figure S7. The Spearman correlation coefficient (SCC) between expression profiles of miRNAs and mRNAs from the BROAD and NCI-60 datasets.** The SCC distributions are similar to PCC in Additional file 1, Figure S6. (A) (D) Solid line: the distribution of SCC between the expression profiles of miRNAs and corresponding predicted targets; dashed line: the distribution of SCC between the expression profiles of miRNAs and non-target genes.


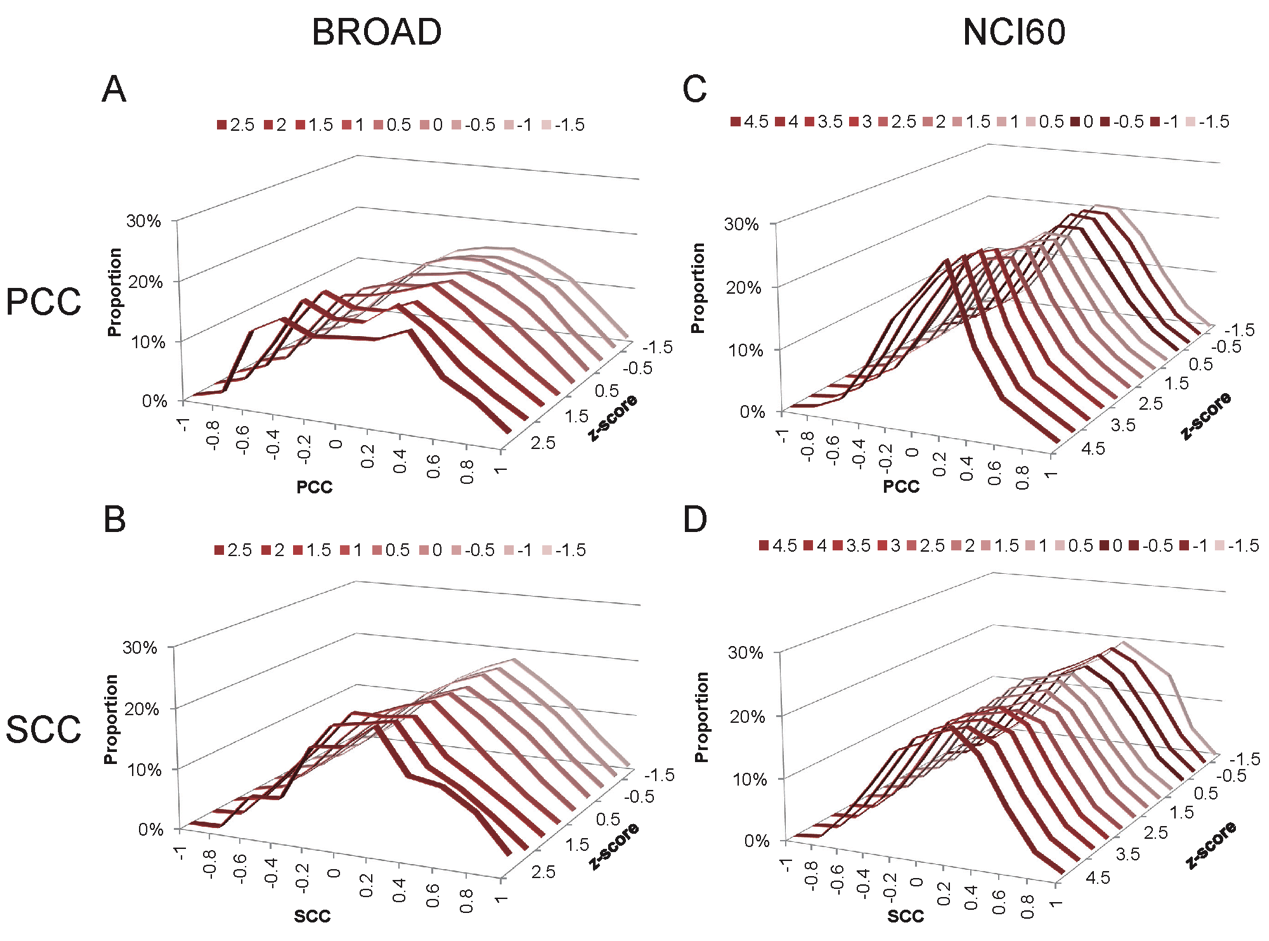


**Figure S8. The distributions of PCC and SCC between expression profiles of miRNAs and non-target mRNAs with different *z*-scores for the BROAD and NCI-60 datasets.**


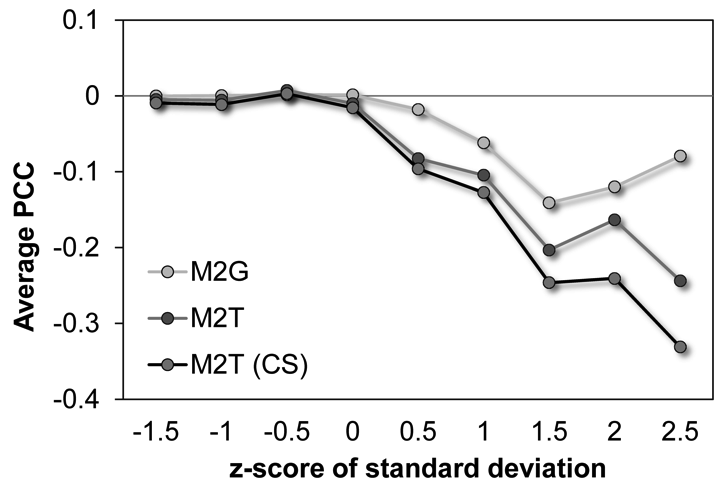


**Figure S9. Relationships between average PCC and Z-score thresholds.** The line charts described the average PCC between the miRNAs and mRNAs with *Z*-scores higher than the given values, from -1.5 to 2.5. Light gray line: miRNAs versus all genes; Dark gray line: miRNAs versus targets; Black line: miRNA versus the targets with lower context scores (more favorable miRNA-target binding).

**Tables**

**Table S1. Activities of the 23 down-regulated miRNA-regulated PINs in normal and tumor tissues.**

| Down-regulated miRNAs |  |  | Normal | | Tumor | |
| --- | --- | --- | --- | --- | --- | --- |
|  | Fold  Change | T-test *p*-value | *p*-value | Activity | *p*-value | Activity |
| miR-143 | 0.0397 | 1.52E-06 | 1 | - | 0.01 | activated |
| miR-29c | 0.0630 | 2.92E-11 | 0.08 | - | 0.01 | activated |
| let-7b | 0.1075 | 8.32E-13 | 0.96 | - | < 0.001 | activated |
| let-7a | 0.1079 | 6.71E-11 | 0.96 | - | < 0.001 | activated |
| miR-29a | 0.1224 | 1.64E-12 | 0.08 | - | 0.01 | activated |
| miR-26a | 0.1436 | 1.08E-10 | 0.21 | - | < 0.001 | activated |
| let-7f | 0.1718 | 1.81E-08 | 0.96 | - | < 0.001 | activated |
| miR-22 | 0.1908 | 4.20E-09 | 0.56 | - | < 0.001 | activated |
| miR-141 | 0.1912 | 6.65E-10 | 0.08 | - | 0.01 | activated |
| miR-142-3p | 0.1930 | 3.10E-05 | 0.35 | - | 0.02 | activated |
| miR-29b | 0.2186 | 4.13E-10 | 0.08 | - | 0.01 | activated |
| miR-148a | 0.2201 | 1.51E-05 | 1 | - | 0.07 | activated |
| miR-768-3p | 0.2990 | 1.04E-05 | 0.12 | - | < 0.001 | activated |
| let-7g | 0.3555 | 3.63E-06 | 0.96 | - | < 0.001 | activated |
| let-7c | 0.3696 | 7.83E-11 | 0.96 | - | < 0.001 | activated |
| miR-200a | 0.3805 | 3.22E-06 | 0.08 | - | 0.01 | activated |
| miR-16 | 0.3378 | 4.59E-10 | 0 | activated | 0.03 | activated |
| miR-145 | 0.0207 | 2.08E-07 | 0.01 | inactivated | 0.2 | - |
| miR-23b | 0.1874 | 2.27E-12 | < 0.001 | inactivated | 0.93 | - |
| miR-27b | 0.4324 | 1.39E-09 | < 0.001 | inactivated | 0.8 | - |
| miR-638 | 0.1357 | 7.04E-07 | 0.93 | - | 0.68 | - |
| miR-200c | 0.1689 | 1.16E-11 | 0.52 | - | 0.28 | - |
| miR-24 | 0.2120 | 2.25E-11 | 0.47 | - | 0.54 | - |

**Table S2. Activities of the 39 unchanged miRNA-regulated PINs in normal and tumor tissues.**

| Unchanged microRNAs | Normal | | Tumor | |
| --- | --- | --- | --- | --- |
|  | *p*-value | Activity | *p*-value | Activity |
| miR-21 | < 0.001 | activated | < 0.001 | activated |
| miR-107 | 0.01 | activated | 0.04 | activated |
| miR-195 | < 0.001 | activated | 0.03 | activated |
| miR-103 | 0.01 | activated | 0.04 | activated |
| miR-15b | < 0.001 | activated | 0.03 | activated |
| miR-194 | 0.91 | - | < 0.001 | activated |
| miR-19b | 0.43 | - | 0.01 | activated |
| miR-324-3p | 0.12 | - | 0.01 | activated |
| let-7d | 0.96 | - | < 0.001 | activated |
| let-7e | 0.96 | - | < 0.001 | activated |
| let-7i | 0.96 | - | < 0.001 | activated |
| miR-34a | 0.74 | - | 0.03 | activated |
| miR-193b | 0.72 | - | 0.03 | activated |
| miR-222 | 0.50 | - | < 0.001 | activated |
| miR-370 | 0.01 | activated | 0.13 | - |
| miR-130a | 0.00 | activated | 0.30 | - |
| miR-23a | < 0.001 | inactivated | 0.93 | - |
| miR-27a | < 0.001 | inactivated | 0.80 | - |
| miR-30d | 0.01 | inactivated | 0.20 | - |
| miR-494 | 0.56 | - | 0.32 | - |
| miR-192 | 1.00 | - | 1.00 | - |
| miR-200b | 0.52 | - | 0.28 | - |
| miR-375 | 1.00 | - | 1.00 | - |
| miR-215 | 1.00 | - | 1.00 | - |
| miR-146a | 1.00 | - | 1.00 | - |
| miR-125b | 0.34 | - | 0.33 | - |
| miR-126 | 1.00 | - | 1.00 | - |
| miR-768-5p | 1.00 | - | 1.00 | - |
| miR-142-5p | 0.33 | - | 0.13 | - |
| miR-575 | 1.00 | - | 1.00 | - |
| miR-106b | 0.73 | - | 0.69 | - |
| miR-214 | 0.36 | - | 1.00 | - |
| miR-25 | 0.81 | - | 0.18 | - |
| miR-572 | 1.00 | - | 1.00 | - |
| miR-31 | 1.00 | - | 1.00 | - |
| miR-223 | 0.08 | - | 0.12 | - |
| miR-93 | 0.73 | - | 0.69 | - |
| miR-106a | 0.73 | - | 0.69 | - |
| miR-451 | 1.00 | - | 1.00 | - |

**Table S3. Receiver operating characteristic (ROC) curves of the 23 down-regulated miRNAs in gastric cancer to classify tumor and normal samples.**

| **miRNAs** | **AUC** | **95% Cl of AUC** | **Overall**  **correct classification (%)** |
| --- | --- | --- | --- |
| **16 oncomirs** | | | |
| miR-29c | 0.831^***^ | 0.687-0.927 | 77 |
| miR-29b | 0.661^*^ | 0.503-0.797 | 66 |
| miR-29a | 0.645 | 0.486-0.783 | 61 |
| miR-200a | 0.502 | 0.348-0.656 | 57 |
| miR-141 | 0.620 | 0.461-0.762 | 64 |
| miR-768-3p | 0.715^**^ | 0.559-0.841 | 70 |
| miR-26a | 0.711^**^ | 0.555-0.837 | 70 |
| miR-142-3p | 0.599 | 0.441-0.744 | 57 |
| miR-22 | 0.589 | 0.430-0.735 | 59 |
| let-7c | 0.655 | 0.497-0.792 | 68 |
| let-7g | 0.661^*^ | 0.503-0.797 | 66 |
| let-7b | 0.671^*^ | 0.514-0.805 | 64 |
| let-7a | 0.645 | 0.486-0.783 | 59 |
| let-7f | 0.618 | 0.459-0.760 | 59 |
| miR-143 | 0.783^***^ | 0.633-0.893 | 75 |
| miR-148a | 0.785^***^ | 0.635-0.894 | 73 |
| **7 down-regulated miRNAs only** | | | |
| miR-16 | 0.851 | 0.361-0.670 | 45 |
| miR-23b | 0.669^*^ | 0.511-0.804 | 66 |
| miR-27b | 0.655 | 0.497-0.792 | 61 |
| miR-145 | 0.822^***^ | 0.678-0.921 | 80 |
| miR-24 | 0.539 | 0.383-0.690 | 39 |
| miR-200c | 0.597 | 0.439-0.742 | 59 |
| miR-638 | 0.680^*^ | 0.522-0.812 | 66 |
| **Combinations**^†^ | | | |
| All 16 oncomirs | 0.981^****^ | 0.886-1.000 | 93 |
| All 7 down-regulated miRNAs | 0.888^****^ | 0.757-0.963 | 82 |

1. These data were obtained by miRNA microarray from 22 gastric cancer patients.

2. AUC, area under the receiver operating curve.

3. Cl, confidence interval.

4. Significant values of AUC, ^*^*P* < 0.05; ^**^*P* < 0.01; ^***^*P* < 0.001; ^****^*P* < 0.0001.

5. ^†^Analyzed by stepwise logistic regression (enter variable if *P* < 0.05; remove variable if *P* > 0.1)

**Table S4. Higher coverage of union genes compared to L0 genes suggests the stronger functional relationship between union genes than L0 genes.**

| Category | Genes | GO Terms | GO Trees | Coverage (%) |
| --- | --- | --- | --- | --- |
| L0 | 173.4 | 24.9 | 7.5 | 44 |
| L1 | 1144.9 | 453.6 | 38.7 | 98 |
| Union | 1318.3 | 479.4 | 40.0 | 99 |

This table shows that the average number of enriched GO terms and trees in which genes, L0, L1, and union of L0 and L1 are involved.

Union: the union set of L0 and L1 genes.

Coverage: the proportion of genes covered by enriched GO functions.

**Table S5. The over-represented functions of oncomir-regulated PINs in gastric cancer.**

| miRNA Family | Enriched Functions |
| --- | --- |
| miR-29abc | Ras protein signal transduction, phosphate metabolism, post-translational protein modification, intracellular signaling |
| miR-141/200a | RNA elongation from RNA polymerase II promoter, transcription initiation from RNA polymerase II promoter, post-translational protein modification, interphase of mitotic cell cycle,  ectoderm development, intracellular signaling |
| miR-768-3p | Negative regulation of cell growth, DNA replication, cell cycling |
| miR-26a | RNA elongation from RNA polymerase II promoter, interphase of mitotic cell cycle, post-translational protein modification, transcription initiation from RNA polymerase II promoter, ectoderm development, intracellular signaling |
| miR-142-3p | Apoptosis, apoptotic mitochondrial changes, regulation of protein homo-/hetero-dimerization activity, regulation of mitochondrial membrane potential, negative regulation of developmental processes |
| miR-22 | Actin cytoskeleton organization and biogenesis, actin filament polymerization, negative regulation of endocytosis, signal transduction |
| let-7/98 | Ras protein signal transduction, anterior/posterior pattern formation, interphase of mitotic cell cycle, apoptosis, negative regulation of gene expression |
| miR-143 | Regulation of chemokine biosynthetic processes, regulation of blood coagulation, blood coagulation, acute-phase response, positive regulation of transcription, wound healing |
| miR-148a | Integrin-mediated signaling, cell-matrix adhesion, blood coagulation, wound healing |

**Table S6.** **Cox proportional hazards regression: significance of clinicopathologic factors on overall survival.**

| **Variable** | **Univariate analysis** | | **Multivariate analysis** | |
| --- | --- | --- | --- | --- |
|  | **HR (95% Cl)** | ***P*** | **HR (95% Cl)** | ***P*** |
| miR-148a^†^ | 0.27 (0.08 to 0.88) | 0.03 | 1.69 (0.61 to 2.77) | 0.002 |
| Stage: early (I + II) vs advanced (III + IV) | 0.10 (0.07 to 0.42) | < 0.001 | 2.43 (0.84 to 4.02) | 0.003 |
| Peritoneal invasion | 0.02 (0.002 to 0.25) | 0.002 | 0.46 (-1.26 to 2.17) | 0.601 |
| Organ invasion | 0.66 (0.19 to 2.34) | 0.518 | -1.04 (-2.05 to -0.02) | 0.047 |
| Vascular invasion | 0.27 (0.1 to 0.69) | 0.006 | 1.71 (-0.38 to 3.79) | 0.11 |

Abbreviation: HR, hazard ratio; 95% Cl, Confidence interval of the estimated HR.

^†^Cutoff = 0.101

**Table S7. Relationship between miR-148a expression levels and clinical factors.**

|  | **No. of Patients** | **%** | **Mean (2-ΔΔCt)** | ***p*-value** |
| --- | --- | --- | --- | --- |
| Fold change (T/N) | 62 |  | 0.524 |  |
| **Gender**^a^ |  |  |  | 0.183 |
| Male | 39 | 63 | 0.415 |  |
| Female | 23 | 37 | 0.710 |  |
| **Stage**^b^ |  |  |  | 0.283 |
| I | 12 | 19 | 0.946 |  |
| II | 14 | 23 | 0.204 |  |
| III | 26 | 42 | 0.528 |  |
| IV | 10 | 16 | 0.459 |  |
| **Depth of tumor invasion**^b^ |  |  |  | 0.279 |
| T1 | 8 | 13 | 1.153 |  |
| T2 | 9 | 15 | 0.390 |  |
| T3 | 43 | 69 | 0.439 |  |
| T4 | 2 | 3 | 0.456 |  |
| **Lymph node metastasis**^b^ |  |  |  | 0.237 |
| N0 | 21 | 34 | 0.235 |  |
| N1 | 25 | 40 | 0.809 |  |
| N2 | 11 | 18 | 0.255 |  |
| N3 | 5 | 8 | 0.650 |  |
| **Distant metastasis**^a^ |  |  |  | 0.043^*^ |
| Negative | 58 | 94 | 0.549 |  |
| Positive | 4 | 6 | 0.238 |  |
| **Lauren classification**^a^ |  |  |  | 0.179 |
| Intestinal-type | 58 | 94 | 0.549 |  |
| Diffuse-type | 4 | 6 | 0.238 |  |
| **Organ invasion**^a^ |  |  |  | 0.013^*^ |
| Negative | 27 | 44 | 0.630 |  |
| Positive | 35 | 56 | 0.415 |  |
| **Vascular invasion**^a^ |  |  |  | 0.305 |
| Negative | 51 | 82 | 0.588 |  |
| Positive | 11 | 18 | 0.230 |  |
| **Peritoneal invasion**^a^ |  |  |  | 0.040^*^ |
| Negative | 17 | 27 | 0.676 |  |
| Positive | 45 | 73 | 0.467 |  |

T: tumor; N: normal. ^a^ *t*-test analysis. ^b^ One-way ANOVA analysis. ^*^*P* < 0.05.

**Methods**

**Correlations between Expression Profiles of miRNAs and Target Genes**

Although miRNAs were reported to cause mRNA degradation of their target genes , we observed no significant changes in the correlation between expression profiles of miRNAs and their predicted target genes across tissues of two independent datasets, BROAD and NCI-60 (Additional file 1, Figure S6A and S6D). This indicates that the predicted target gene set may not be able to reflect the expression correlations between miRNAs and corresponding targets across tissues. Therefore, we speculated that, if some miRNAs are over-expressed greatly in some tissues, its specific target genes should be down-regulated substantially in the same tissues. In other words, a reverse correlation in expression profiles between a miRNA and its predicted target will increase the confidence of the conditional miRNA-target gene interaction. To manifest this, we selected the miRNAs and corresponding targets both with large expression variation across tissues, great standard deviation of expressions across tissues, and investigated their expression correlation. The z-score of the standard deviation of each expression profile across tissues was calculated to quantify the expression variation of each miRNA or gene. Pearson correlation coefficient (PCC) between the expression profiles of each pair of miRNA and target gene was used to evaluate their expression correlation. In both datasets, the average PCC was inversely proportional to the expression variation of miRNAs and their target genes (Additional file 1, Figure S6B and S6E). Furthermore, this trend was consistent and observed across different datasets from both normal (BROAD, Additional file 1, Figure S6B) and cancer tissues (NCI-60, Additional file 1, Figure S6E). To further confirm this result, the PCC distributions between miRNAs and predicted target genes with different z-score categories were investigated. As can be seen for the BROAD and NCI-60 dataset, the area of distribution for negative values of PCC (PCC < 0) correlated positively with the z-score (Additional file 1, Figure S6C and S6F). Consequently, the above results suggest that miRNA expression levels tend to correlate negatively with the expression levels of corresponding targets when both are highly variable across tissues. To further confirm the conclusion we suggested, PCC was substituted with Spearman correlation coefficient (SCC) to repeat the above analysis procedure, and consistent results were observed (Additional file 1, Figure S7). On the other hand, we also investigated miRNAs and non-target mRNAs, but did not observe such correlations between them (Additional file 1, Figure S8).

To examine if the PCC and *z*-score has any intrinsic relationships, we repeated this analysis by using miRNAs and all genes instead of miRNAs and targets. The result was shown in Additional file 1, Figure S9 (light gray line, M2G). Indeed, there exists some intrinsic trend toward negative PCC as *z*-score increases. However, miRNA versus target mRNAs show stronger negative correlation (dark gray, M2T). By selecting more confident miRNA targets using the context scores provided by TargetScan Human 5.1, we observed even stronger negative correlation (black, M2T_CS). TargetScan uses the context score to evaluate each predicted miRNA-target binding. A lower context score means more favorable miRNA-target binding. Here, we selected the miRNA-target pairs with their context scores lower than the average among the miRNA to all its targets. These results show that, although there exists intrinsic bias between PCC and *z*-score, the negative correlation between PCC and *z*-score of miRNA and targets is stronger than an artifact.

Due to the presence of the primary TF regulation, expression of a miRNA target may not always co-vary, in the reverse direction, with its miRNA regulator across different tissues or conditions. Our results suggested that a reverse correlation in expression profiles between a miRNA and its predicted target will increase the confidence of the predicted target being more likely to be a true target, or in other words, increase the confidence of the conditional miRNA-target interaction.

**Two miRNA and mRNA Expression Datasets**

Various tissue types from two datasets, BROAD (non-tumor tissues) and NCI-60 (tumor tissues) , were used to investigate the correlation between expression profiles of miRNAs and corresponding targets. The “BROAD” dataset was obtained from <http://www.broadinstitute.org/publications/broad856> and included both miRNA and mRNA expression profiles . In this study, eight normal tissues, including colon, pancreas, kidney, bladder, prostate, uterus, lung, and breast, were used because the expression profiles of the miRNA for these tissues were complete. The NCI-60 dataset was downloaded from the ArrayExpress database <http://www.ebi.ac.uk/microarray-as/ae/> and also included both miRNA and mRNA expression profiles. 9 tumor samples from breast, central nervous, colon, leukemia, melanoma, non-small cell lung, ovarian, prostate, and renal cancer were used. The accession numbers were E-MEXP-1029 and E-GEOD-5720 , respectively.

**Expression Variations of miRNA and mRNA**

To evaluate the expression variations (variability or divergence) of miRNA and mRNA, the standard deviation (σ) of expression levels of each miRNA, or mRNA, across all tissues studied was calculated. The standard deviation (σ) of *X*, miRNA or mRNA, is defined as:

,

where *n* is the number of studied tissues, is the expression level of *X* in tissue *i*, and represents the average expression level of *X* across all tissues. Each miRNA (or mRNA) has a respective σ(*X*). Since the expression levels of miRNA and mRNA are in different ranges, we used *z*-score for standardization of σ(*X*). With all the miRNAs, we calculated the mean () and the standard deviation () of their σ(*X*) distribution. Then we defined the *z*-score of σ(*X*) for each miRNA as:

.

The *Z*-score represents the relative expression variation (divergence or variability) across tissues of each miRNA among all the measured miRNAs. Similarly, we calculated the *Z*-score of each mRNA separately, to quantify the relative expression variation (divergence or variability) across tissues of each mRNA among all the measured mRNAs.

**Tissue Specimens**

RNA from paired normal and tumor specimens from gastric cancer patients were extracted for miRNA microarray hybridization. The paired tissue specimens were dissected within 30 minutes of gastrectomy and frozen in liquid nitrogen tank. Healthy mucosa samples were taken from areas of grossly normal mucosa located at least 3 cm from the tumor border.

The criteria for curative resection included the complete removal of primary gastric tumor, D2 dissection of regional lymph nodes and absence of macroscopic tumor remaining after surgery. No other previous or concomitant primary cancer was present. No patient had received chemotherapy and radiotherapy before surgery. Clinicopathologic factors including age, sex, gross types of tumors (Borrmann classification), histologic types of tumors (Lauren classification), depth of tumor invasion, lymph node status, direct invasion of organs such as duodenum, esophagus, liver, pancreas, and mesocolon, and distant metastasis documented histologically were reviewed and stored in a patients’ database. The patients were followed up for 2 to 140 months after surgery. The follow-up intervals were calculated as survival intervals after surgery.

**MicroRNA Microarray**

100 ng of total RNA were dephosphorylated with 11.2 units of calf intestine alkaline phosphatase (GE Healthcare Life Sciences, Sweden) for 30 min at 37°C. The reaction was terminated after dephosphorylation at 100°C for 5 min and then immediately frozen. 5μL of DMSO was added and samples were heated to 100°C for 5 min and immediately frozen again. Ligase buffer and BSA were added and ligation was performed with 50μM pCp-Cy3 and 28μL T4 RNA ligase (GE Healthcare Life Sciences, Sweden) at 16°C for 2h. The labeled miRNAs were desalted with MicroBioSpin6 columns (BioRad, USA). 2X hybridization buffer (Agilent Technologies, USA) was added to the labeled mixture to a final volume of 45μl. The mixture was heated for 5 min at 100°C and immediately frozen. Each 45μl sample was hybridized onto Agilent human miRNA Microarray (Agilent Technologies, USA) at 55°C for 20h. After hybridization, slides were washed at room temperature in Gene Expression Wash Buffer 1, then in Gene Expression Wash Buffer 2 (Agilent Technologies) for 5 min, respectively. Slides were scanned using an Agilent microarray scanner (Agilent Technologies, model G2565A) at 100% and 5% sensitivity settings. Feature Extraction (Agilent Technologies) software version 9.5.3 was used for image analysis. We have submitted the miRNA microarray data to the GEO database and the series record is GSE28700.

**Activity of miRNA-regulated PIN**

To investigate the activity of miRNA-regultaed PIN, the number of co-expressed protein-protein interactions (CePPIs) within the miRNA-regulated PINs was considered. The cutoff of Pearson correlation coefficient (PCC) was determined by the comparison of the PCC distribution between PPI pairs and all paired genes. In both tumor and normal conditions, we observed that the proportion of PPI PCC became higher than that of all paired PCC when PCC was higher than 0.3 (Additional file 1, Figure S5). Additionally, as the p-value of PCC <= 0.05, PCC for tumor and normal was about 0.36 and 0.32 respectively. Therefore, paired genes with significant positive PCCs (*P*-value of PCC <= 0.05; tumor: PCC > 0.36; normal: PCC > 0.32) were considered as co-expressed under a specific biological condition, i.e. gastric cancer or normal tissues. Protein-protein interactions (PPIs) between proteins encoded by co-expressed gene pairs were defined as CePPIs. Large numbers of CePPIs involved within the miRNA-regulated PIN is associated with greater activity. Fisher's exact test was applied to test the numbers of CePPIs involved in the miRNA-regulated PIN and determine whether the activity of the miRNA-regulated PIN was significant (*P* ≤ 0.05). The reference set of Fisher's exact test consisted of PPIs which were formed by proteins encoded by target genes of these miRNAs and their interacting partners in the human PIN. We further classified the miRNA-regulated PINs into two groups; activated and inactivated, according to the significance of the activities of miRNA-regulated PINs. For example, miRNA-regulated PINs with significantly over-represented CePPIs in tumor (normal) were considered as “activated in tumor (normal)” while those with significantly under-represented CePPIs in tumor (normal) were considered as “inactivated in tumor (normal)”.

**Investigation of the Functional Roles of miRNA**

To investigate the functional roles of miRNAs, the predicted target genes of miRNAs was integrated into and analyzed within PIN. We defined L0 genes as the predicted target genes of miRNAs and L0 proteins were encoded by L0 genes, while L1 proteins are interacting partners of L0 proteins in the human PIN. The functional roles that miRNAs play within the PIN constructed by L0 and L1 proteins were predicted as significantly over-represented Gene Ontology (GO) terms . BiNGO , a Cytoscape plug-in, was used to determine which GO terms were significantly over-represented (Hypergeometric test *P* ≤ 0.001) in miRNA-regulated PINs. A Hypergeometric test was performed to determine whether the GO terms were significantly over-represented. The hypergeometric distribution is described as:

,

where *X* denotes the evaluated functional category in GO, *N* (*m*) represents the number of GO annotated genes participated in HPRD protein interaction network (PIN) and n represents the number of genes which are annotated as the evaluated GO functional category in HPRD PIN. Thus, this formula calculates the probability of the evaluated functional category covering *k* genes in that network. The calculated *P*-value was then adjusted by applying the Benjamini and Hochberg multiple testing procedures to control the false discovery rate (FDR) . In order to obtain more specific functions of miRNA-regulated PINs, only GO terms with a GO level greater than or equal to 5 were considered to be potential biological functions of miRNA-regulated PINs. In addition, we defined a GO tree as a weakly connected component in a directed graph, in which nodes represent GO terms and links represent the relationships between nodes to summarize a group of enriched GO terms.

**qRT-PCR for miRNA**

Samples were analyzed by SDS-PAGE to confirm that there was no RNA degradation. The concentration of total RNA was quantified using a ND-1000 spectrophotometer (NanoDrop Technologies) and diluted to 5ng/μl for further analysis. RNA samples were mixed with miRNA-specific primers and a PCR reaction was performed for 30 minutes at 16°C, 30 minutes at 42°C, and 5 minutes at 85°C. cDNA products were mixed with miRNA-specific assay probes and incubated for 10 minutes at 95°C, 15 seconds at 95°C and 1 minute at 60°C for a total of 40 cycles using a 7300 real-time PCR system (Applied Biosystems). U6 small nuclear RNA was measured using the same method and was used for normalization.

**Invasion Assays** **Using Boyden Chamber Assays**

AGS (2.5 x 10^4^ cells), SC-M1 (1 x 10^5^ cells) and MKN-45 cells (2 x 10^5^ cells) in 100μl medium were added to the upper chamber and cells were fixed, stained, viewed and counted by a light microscope (Olympus) after 48hr in culture. Each experiment was repeated three times.

**Cell Adhesion Assay**

5 x 10^3^ cells per 100μl were seeded in each well and incubated at 37°C for 15 minutes. After incubation, non-adhesive cells were washed with PBS and the cells were fixed with 4% formaldehyde at room temperature for 20 minutes. Cells were washed and stained with 0.05% crystal violet. Cells were viewed and counted using a microscope (Olympus). Each experiment was repeated three times.

**Luciferase Reporter Assay**

A luciferase assay was used to analyze the relationship between miR-148a and **plasminogen activator inhibitor 1 (**PAI-1), **guanine nucleotide exchange factor** VAV2 (VAV2), integrin alpha-5 (ITGA5) and **integrin beta-8 (**ITGB8). The target sites in 3’-UTRs of these genes were inserted into the Luciferase reporters as described in the Additional file 1, Methods section. A luciferase assay was performed to analyze the relationship between miR-148a, PAI-1, VAV2, ITGA5 and ITGB8. Luciferase reporters containing the target sites in 3’-UTRs of these genes were constructed using the following oligonucleotides: for PAI-1: (sense) 5’-AATGCGAGCTCTTTTGATTTTGCACTGGACGGTGACGTGCTCAGCAAGCTTAATGC-3’ and (antisense) 5’-GCATTAAGCTTGCTGAGCACGTCACCGT CCAGTGCAAAATCAAAAGAGCTCGCATT-3’; for VAV2: (sense)
5’-AATGCGAGCTCTGGTTTTTGCACTGCAGCTCAGCAAGCTTAATGC-3’ and (antisense) 5’-GCATTAAGCTTGCTGAGCTGCAGTGCAAAAACCAGAGCT CGCATT-3’; for ITGA5: (sense) 5’-AATGCGAGCTCCCTGCCAGCTGCACTGAT GCTGGCTCAGCAAGCTTAATGC-3’ and (antisense) 5’-GCATTAAGCTTGC TGAGCCAGCATCAGTGCAGCTGGCAGGGAGCTCGCATT-3’ and for ITGB8:
(sense) 5’-AATGCGAGCTCGCACTGAGCTCAGCAAGCTTAATGC-3’ and (antisense) 5’-GCATTAAGCTTGCTGAGCTCAGTGCGAGCTCGCATT-3’. The oligonucleotides were annealed and the product was digested with HindIII and SacI (New England Biolabs Ltd., Ipswich, MA, USA) and cloned into the pMIR-REPORT luciferase expression vector (Ambion, Austin, TX). Positive clones were digested with BlpI restriction enzyme for screening (New England Biolabs Ltd., Ipswich, MA, USA) and the selected clones were verified by sequencing (Mission Biotech Co. Ltd). AGS cells cultured in a 24-well plate (8 x 10^4^ cells per well) were transfected with 200ng each of luciferase and β-galactosidase luciferase reporter vectors and co-transfected with 50μM miR-148a precursor or miR-148a inhibitor. After transfection for 48hr, firefly luciferase and β-galactosidase were measured sequentially with a Spectramax M5 ELISA reader (Molecular Devices Corporation) using the Dual-Light system (Applied Biosystems) according to the manufacturer’s protocol. β-galactosidase activity was used for normalization of transfection efficiency.

**Immunoblotting**

Proteins were transferred onto PVDF membranes (Immobilon-P membrane; Millipore Corp, Bedford, MA), incubated with antibodies against PAI-1 (Santa Cruz Biotechnology, Santa Cruz, CA), VAV2 (Epitomics Biotechnology, Burlingame, CA), ITGA5 (Santa Cruz Biotechnology, Santa Cruz, CA) and ITGB8 (Santa Cruz Biotechnology, Santa Cruz, CA) and visualized with the ECL detection kit (Pierce, Boston Technology, Woburn, MA).

**Statistical Analysis**

To analyze the correlation between miRNAs and their corresponding clinical outcomes, patients were divided into different groups based on clinical and pathologic parameters. Student’s t-test was used to compare the differences between two groups, while comparisons between multiple groups were performed using one-way ANOVA analysis (SAS 9.1). Correlations between numeric variables were analyzed using MedCalc 9.0, where the data are shown as a correlation coefficient with a *P*-value. A Kaplan-Meier overall survival curve was used to compare miRNAs with patient survival, where the analytic method used was log-rank test. Cox proportional hazards regression models determined their prognostic independence of clinical factors. In addition, the difference in D values between high and low groups for the 23 down-regulated miRNAs (median was used as a cut-off value) was calculated by paired Wilcoxon rank sum test. The alpha level of significance was defined as *P* < 0.05.

**References**

1. Bartel DP: **MicroRNAs: genomics, biogenesis, mechanism, and function.** *Cell* 2004, **116:**281-297.

2. Lu J, Getz G, Miska EA, Alvarez-Saavedra E, Lamb J, Peck D, Sweet-Cordero A, Ebert BL, Mak RH, Ferrando AA, et al: **MicroRNA expression profiles classify human cancers.** *Nature* 2005, **435:**834-838.

3. Blower PE, Verducci JS, Lin S, Zhou J, Chung JH, Dai Z, Liu CG, Reinhold W, Lorenzi PL, Kaldjian EP, et al: **MicroRNA expression profiles for the NCI-60 cancer cell panel.** *Mol Cancer Ther* 2007, **6:**1483-1491.

4. Shankavaram UT, Reinhold WC, Nishizuka S, Major S, Morita D, Chary KK, Reimers MA, Scherf U, Kahn A, Dolginow D, et al: **Transcript and protein expression profiles of the NCI-60 cancer cell panel: an integromic microarray study.** *Mol Cancer Ther* 2007, **6:**820-832.

5. Ashburner M, Ball CA, Blake JA, Botstein D, Butler H, Cherry JM, Davis AP, Dolinski K, Dwight SS, Eppig JT, et al: **Gene ontology: tool for the unification of biology. The Gene Ontology Consortium.** *Nat Genet* 2000, **25:**25-29.

6. Maere S, Heymans K, Kuiper M: **BiNGO: a Cytoscape plugin to assess overrepresentation of gene ontology categories in biological networks.** *Bioinformatics* 2005, **21:**3448-3449.

7. Shannon P, Markiel A, Ozier O, Baliga NS, Wang JT, Ramage D, Amin N, Schwikowski B, Ideker T: **Cytoscape: a software environment for integrated models of biomolecular interaction networks.** *Genome Res* 2003, **13:**2498-2504.

8. Benjamini Y, Yekutieli D: **The control of the false discovery rate in multiple testing under dependency.** *Annals of Statistics* 2001, **29:**1165-1188.
